# Bone Marrow Failure Syndromes, Overlapping Diseases with a Common Cytokine Signature

**DOI:** 10.3390/ijms22020705

**Published:** 2021-01-12

**Authors:** Valentina Giudice, Chiara Cardamone, Massimo Triggiani, Carmine Selleri

**Affiliations:** 1 Department of Medicine, Surgery and Dentistry “Scuola Medica Salernitana”, University of Salerno, Baronissi, 84081 Salerno, Italy; vgiudice@unisa.it (V.G.); ccardamone@unisa.it (C.C.); cselleri@unisa.it (C.S.); 2 Clinical Pharmacology, University Hospital “San Giovanni di Dio e Ruggi D’Aragona”, 84131 Salerno, Italy; 3 Hematology and Transplant Center, University Hospital “San Giovanni di Dio e Ruggi D’Aragona”, 84131 Salerno, Italy; 4 Internal Medicine and Clinical Immunology, University Hospital “San Giovanni di Dio e Ruggi D’Aragona”, 84131 Salerno, Italy

**Keywords:** cytokines, bone marrow failure syndromes, aplastic anemia, myelodysplastic syndromes

## Abstract

Bone marrow failure (BMF) syndromes are a heterogenous group of non-malignant hematologic diseases characterized by single- or multi-lineage cytopenia(s) with either inherited or acquired pathogenesis. Aberrant T or B cells or innate immune responses are variously involved in the pathophysiology of BMF, and hematological improvement after standard immunosuppressive or anti-complement therapies is the main indirect evidence of the central role of the immune system in BMF development. As part of this immune derangement, pro-inflammatory cytokines play an important role in shaping the immune responses and in sustaining inflammation during marrow failure. In this review, we summarize current knowledge of cytokine signatures in BMF syndromes.

## 1. Introduction

**Figure 1:**
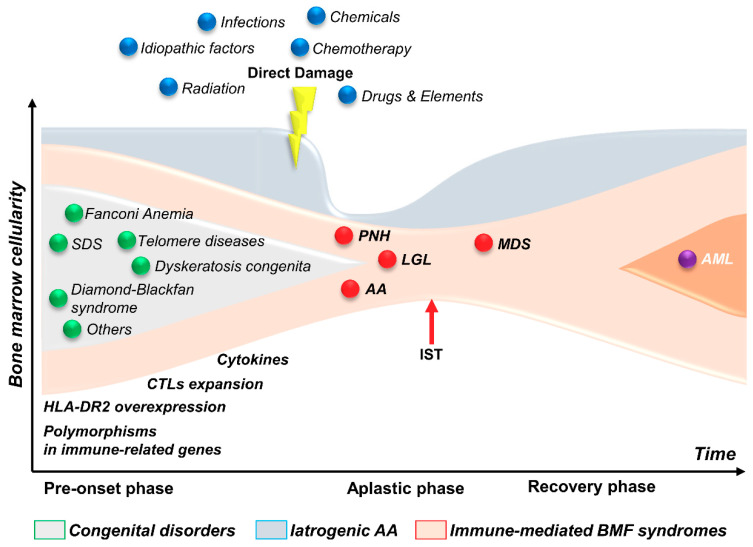
Bone marrow failure (BMF) syndromes’ pathophysiology. BMF syndromes are characterized by empty bone marrow and peripheral blood cytopenia(s), and can be divided in congenital disorders, iatrogenic aplastic anemia (AA), and immune-mediated BMF. In congenital disorders, such as Shwachman–Diamond syndrome (SDS) or Fanconi anemia, hematopoietic stem cells (HSCs) harbor mutations in genes important for normal hemopoiesis, which becomes insufficient over time in maintaining normal ranges of circulating cells. In iatrogenic AA, HSCs are directly damaged by external stressors, such as chemicals and radiation. In immune-mediated BMF, dysregulated immune responses can cause an autologous immune attack of cytotoxic T lymphocytes (CTLs) against HSCs or can suppress hemopoiesis through changes in BM microenvironment. Clinical presentation of BMF syndromes differs among diseases; however, immunosuppressive therapies (ISTs) can often restore bone marrow cellularity, which is one of the major pieces of evidence for the immune-mediated pathogenesis in BMF. Abbreviations. PNH, paroxysmal nocturnal hemoglobinuria; LGL, T-large granular lymphocyte leukemia; AA, acquired aplastic anemia; MDS, myelodysplastic syndromes; AML, acute myeloid leukemia.

Bone marrow failure (BMF) syndromes are a heterogeneous group of non-malignant constitutional and acquired hematological diseases characterized by uni- or multi-lineage marrow and or peripheral blood cytopenia(s), regardless the presence of any other disorder that may affect marrow function [1,2,3,4]. BMF in constitutional syndromes is caused by inherited germline mutations occurring in the hematopoietic stem cell (HSC) compartment or in other hematopoietic stem and progenitor cells (HSPCs), resulting in specific diseases such as Fanconi anemia (FA), dyskeratosis congenita (DKC), Shwachman–Diamond syndrome (SDS), congenital amegakaryocytic thrombocytopenia, and neutropenia (Kostman Disease), as well as familial telomerase diseases. Conversely, acquired BMF syndromes result from extrinsic direct and indirect damages on the HSC pool due to chemical agents, drugs, and different viruses (Figure 1) [1]. However, the indirect injury of HSC is primarily supported by immune effector mechanisms, which may also be triggered by viruses or by drug metabolites. Regardless the type of initial injury on the bone marrow (BM), constitutional and acquired BMF syndromes share qualitative and/or quantitative disfunctions of HSCs or their progenies, which affect their self-renewal ability [2,3,4]. Self-renewal is a hallmark of stemness that allows long-term maintenance of a complex process called hematopoiesis, leading to differentiation and maturation of mature circulating cells. Hematopoiesis is regulated by a complex network between hematopoietic and stromal cells, as well as several soluble and membrane-bound cytokines within and outside the hematopoietic niches [1,2,3,4,5]. Derangement in interaction and cooperativity between cellular and cytokine activities has been widely reported in different acquired BMF syndromes, such as acquired aplastic anemia (AA), hypoplastic myelodysplastic syndromes (hMDS), and chronic T and natural killer (NK) granular lymphocyte disorders.

In this review, we provide an update of the main cytokine abnormalities involved in key relevant pathways of acquired BMF syndromes sharing similar immune-mediated pathogenic mechanisms.

## 2. Acquired Aplastic Anemia

**Figure 2:**
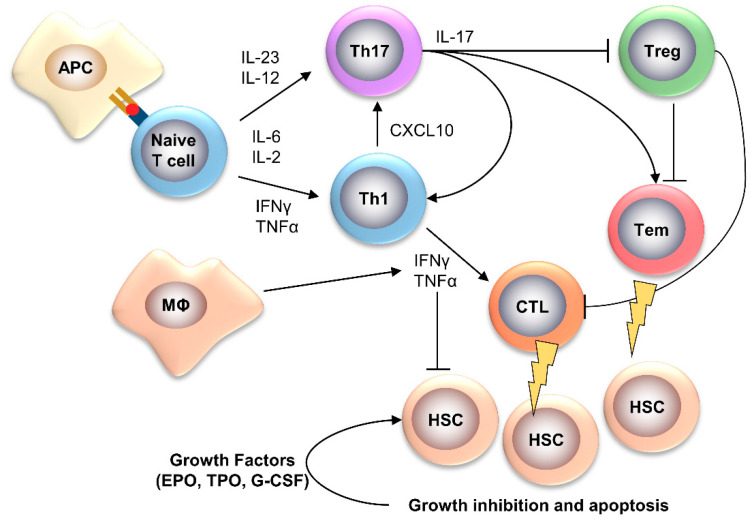
Pathophysiology of AA. In AA, an unknown antigen might trigger a T helper (Th)1 immune response, causing the expansion of cytotoxic T lymphocytes (CTLs), which directly kill hematopoietic stem cells (HSCs) [2,6,7,8]. Chronic antigen exposure might also polarize naïve T cells toward the Th17 phenotype, leading to expansion of effector memory T cells (Tem) with cytotoxic activities and inhibition of T regulatory cells (Treg) [2]. Several interleukins (ILs) and cytokines, such as interferon (IFN)-gamma and tumor necrosis factor (TNF)-alpha, are involved in immune response polarization and direct growth inhibition of HSCs, which leads to increased levels of growth factors for maintaining normal hemopoiesis [8]. APC, antigen-presenting cells; MΦ, macrophage; CXCL, C-X-C motif chemokine; G-CSF, granulocyte colony-stimulating factor; TPO, thrombopoietin; EPO, erythropoietin.

AA is a usually sporadic and immune-mediated BMF syndrome, likely caused by an autologous immune attack against HSPCs, and hematological recovery of blood counts after immunosuppressive therapies (ISTs) is one of the strongest pieces of evidence for the immune-mediated pathogenesis [2]. Cytotoxic T cells (CTLs) play a pivotal role in BM destruction, and type I interferons (IFNs) polarize the immune system toward T helper (Th)1 responses [2,6,7,8]; however, other T cell subsets and cytokines are involved in AA pathogenesis [2]. Oligoclonal expansion of CD8^+^CD28^−^ T cells and effector memory CD8^+^CD28^−^CD57^+^ T lymphocytes is frequent in AA and suggests an antigen driven mechanism of T-cell activation [9,10,11]. Immunodominant clones can be highly enriched in effector memory CD8^+^CD28^−^CD57^+^ T cells, and related CDR3 sequences are private to AA, while they are shared between disease and healthy subjects, suggesting the existence of common epitopes [9]. T regulatory cells (Tregs) are also decreased in AA and their ability to suppress autoreactive clones is reduced, while Th17 cells, associated with autoimmune disorders, are frequently increased, are correlated to disease severity, and are inversely related to the number of CD4^+^CD25^high^FoxP3^+^ Tregs (Figure 2) [12].

### 2.1. Th1 Cytokines

Several cytokines and chemokines are deregulated during AA as the consequence of specific T cell subset expansion or as a direct cause of immune response polarization and BM growth inhibition (Table 1) [13]. In the Th1-mediated immune response, circulating and BM levels of IL-12 and IL-23 are increased in AA and are related to disease severity [14]. IL-12 is mainly secreted by activated dendritic cells and macrophages. IL-12 and IL-23 share a common subunit, p40, and induce IFN-γ production in T and NK cells and polarization of naïve T lymphocytes into Th1 type [15,16,17,18]. IL-18, an IL-1 family member, cooperates with IL-12 in Th1 polarization, CTL activation, and IFN-γ production [19,20,21,22]. IL-18 and IL-18 binding protein serum levels are increased in AA at diagnosis compared with healthy subjects [23], and their concentrations are higher in non-responder patients compared with responders. IL-18 can modulate in vitro gene expression on the BM compartment, especially on CD34^+^ hematopoietic stem cells (HSCs), which show the highest surface expression of the IL-18 receptor compared with other BM populations. However, by knocking down IL-18 or IL-18R, BMF did not ameliorate in mouse models, suggesting a dispensable role of this cytokine in AA pathogenesis [23]. Upon stimulation and differentiation, Th1 cells can produce IL-2 and IFN-γ and activate macrophages and CTLs. Accordingly, IL-2 plasma levels in the BM and in the peripheral blood are increased and related to disease severity [24]. Similarly, IFN-γ and TNF-α BM plasma levels are elevated and decline after recovery [25]; however, circulating TNF-α might be not increased in AA compared with healthy subjects [26]. IFN-γ and TNF-α are historically implicated in AA pathogenesis [8,27]; nevertheless, their effects on HSPC growth and immune system regulation might be different. Exogenous and stromal cell-produced IFN-γ inhibits HSPC growth and reduces self-renewal of HSCs, probably impairing TPO signaling pathways. In addition, IFN-γ directly suppresses erythropoiesis by blocking HPSCs at the earliest stages of differentiation, and likely by inducing IL-15 production, which can also directly suppress hematopoiesis [28,29,30,31]. BM growth is also reduced because of increased apoptosis through induction of Fas expression on HSPCs, which facilitates apoptosis via Fas/Fas ligand (FasL), and by inducing nitric oxide synthase (NOS) and production of nitric oxide [30,32]. Moreover, chronic inflammation causes increased expression of IFN-γ in BM and lymphocytes, particularly in highly IFN-γ producing T-bet^+^ cells [32]. TNF-α is increased in AA and upregulation of its receptors has been described on human BM cells, as well as increased frequency of TNF-α^+^ BM macrophages [33,34,35]. In BMF mouse models, injection of lymph node TNF-α^−/−^ cells into pre-irradiated CByB6F1 recipients does not prevent BMF development, as well as depletion of TNF-α receptor genes in recipient mice [33]. However, depletion of TNF-α producing macrophages in recipient mice abrogates BMF by blocking T cell migration into the BM and by reducing circulating levels of IFN-γ and TNF-α through induction of the master transcriptional regulator Tbx21, which modulates gene expression in CD4^+^ and CD8^+^ T cells [33]. CXCL10 is also elevated in the plasma of AA patients at diagnosis compared with AA patients who achieved a complete remission and healthy subjects [36]. Upon CXCL10 stimulation in vitro, Th1 cell number and Th1/Th2 ratio are higher in AA with decreased Th2 proportion compared with healthy controls [36].

### 2.2. Th17 Cytokines

Th17/Treg deregulation is also linked to modification in related cytokine concentrations in both plasma and BM microenvironment. The plasma concentration of IL-17A is increased in AA and correlates with disease severity and the number of CD4^+^CXCR4^+^ T-cells [12,37]. IL-21, a pro-inflammatory cytokine elevated in several autoimmune disorders, shows increased plasma levels in AA, as well as IL-21-producing CD4^+^ T cells in the BM that are positively correlated with Th17 frequency and negatively correlated with Treg number [38]. Increased CCL20 levels in AA might be also related to a Th17 skewing of immune responses, and increased BM levels might function as chemoattractant of Th17 to BM niche [39]. IL-35 is a heterodimeric member of the IL-12 family composed by IL-12 p35 and IL-27 Epstein–Barr virus-induced protein 3 (EBI3) subunits, and might act as an immunosuppressive cytokine mainly released by Tregs [40,41,42]. IL-35 can inhibit polarization of naïve CD4^+^ T cells into Th1 and Th17, thus reducing related Th1 and Th17 cytokines while inducing a potent Treg population (iTR35) and B regulatory cell expansion [40,43]. In AA, IL-35 plasma levels are decreased and correlated to disease severity, and patients in complete remission have similar levels of those in healthy subjects, suggesting a role of IL-35 in maintaining immune tolerance during BMF [40]. Similarly, in children, IL-35 is decreased at diagnosis and its normalization in the first 28 days after starting IST is associated with prolonged response to therapy [44].

### 2.3. Growth Factors

HSPC growth inhibition and apoptosis determine the release of growth factors to restore normal hemopoiesis. Erythropoietin (EPO), thrombopoietin (TPO), and granulocyte-colony stimulating factor (G-CSF) are required for differentiation and maturation of HSCs into red blood cells, platelets, and granulocytes [45], and their circulating levels are fine tuned to maintain peripheral blood cell levels in physiological ranges. Therefore, plasma levels of EPO, TPO, and G-CSF are significantly increased in AA because of the BMF [26,45,46]. Interestingly, EPO and G-CSF levels dramatically decline and normalize after IST initiation in patients who achieved a complete remission, while TPO levels are persistently high even in responders with stable blood counts after years from IST and/or eltrombopag, a TPO agonist, treatments [45]. TPO is removed from circulation through MPL (myeloproliferative leukemia protein)-mediated internalization and degradation in platelets; therefore, thrombocytopenia in AA determines reduced TPO clearance from peripheral blood, leading to increased circulating levels of this growth factor [46,47,48,49]. However, MPL circulating levels might be persistently reduced in AA patients after 6 months of IST, regardless of responsiveness to therapy [46]. Circulating EPO concentrations are positively correlated with plasma GDF-15, the growth differentiation factor-15, a member of the transforming growth factor-β family involved in iron homeostasis [50]. Indeed, GDF-15 levels are also positively correlated with serum iron and transferrin saturation levels, and percentage of sideroblasts in the BM, while they are negatively correlated with hepcidin levels [50,51]. 

### 2.4. BM Environment 

BM mesenchymal stem cells (BM-MSCs) might be involved in the pathogenesis of AA, because MSCs can differentiate in distinct types of stromal cells that support hematopoiesis and regulate immune cells in the BM niche [52,53,54,55,56]. BM-MSCs have reduced ability to suppress proliferation and differentiation of CD4^+^ cells, and TNF-α and IFN-γ production in AA while inducing Treg polarization without affecting IL-4, IL-10, or IL-17 production. In addition, BM-MSCs themselves show impairment in morphology and multi-lineage differentiation ability, but not in their immunophenotypes [57]. Indeed, establishment efficiency of long-term BM-MSCs from AA patients is lower than that of healthy subjects, and cells have impaired adipogenic differentiation ability with morphologic abnormalities and reduced expression of insulin-like growth factor (IGF)-1, as well as reduced osteogenic differentiation [58]. MSCs in AA show differentially expressed genes compared with MSCs from healthy subjects, and genes are involved in immunoregulation and cellular processes. Other highly expressed genes are Th1, Th2, and Th17 differentiation-associated and inflammation-related genes. In addition, abnormal splicing is also documented and involved genes are related to oncogenesis, metabolism, and other signaling pathways such as mTOR (mammalian target or rapamycin) and Wnt [52,53,54,55,56,57,58].

## 3. hMDS

**Figure 3:**
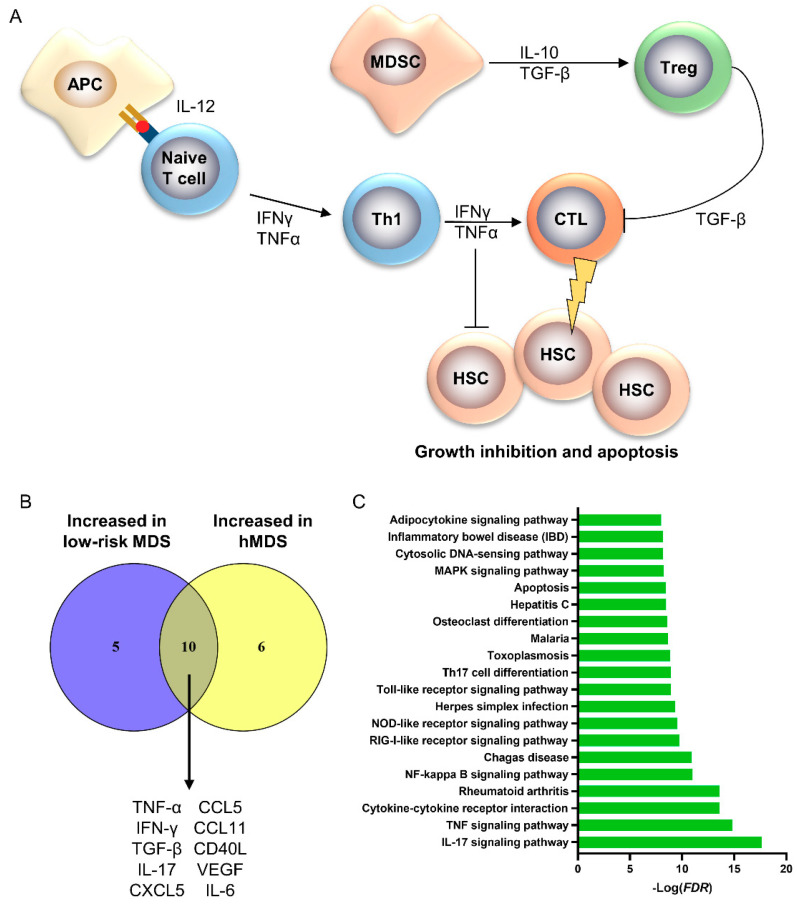
Pathophysiology of hypoplastic myelodysplastic syndrome (hMDS). (**A**) In hMDS, naïve T cells differentiate into Th1 cells, causing CTL activation, which directly kills HSCs [61]. Myeloid derived suppressor cells (MDSC) can also induce expansion of Treg, especially in high-risk MDS. IFN-γ, TNF-α, and tumor growth factor-beta (TGF-β) drive in immune response polarization and direct growth inhibition of HSCs [66,70,71,72,76]. (**B**) Reported cytokines increased in low-risk MDS and hMDS were interpolated using Venn’s diagram, and shared cytokines (*n* = 10) were used for protein pathway analysis to identify common pathways in BMF disease pathophysiology. (**C**) The top 20 related pathways are reported.

hMDS are characterized by BM hypocellularity and peripheral blood cytopenia(s) resembling AA, while clinically overlapping with normo-/hypercellular MDS (NH-MDS) showing dyspoiesis, chromosomal abnormalities, and increased risk of acute myeloid leukemia (AML) [1,59,60]. Differential diagnosis is often challenging because of the lack of specific clinical and molecular features in hMDS. Recurrent genetic and epigenetic alterations are found between hMDS, NH-MDS, and AA at different frequencies without any statistical significance. Indeed, trisomy 8, trisomy 1q, 20q deletion, or monosomy 7 can be found in both hMDS and AA, as well as *RAS*, *AML1*, or *JAK2* mutations in NH-MDS and hMDS [60,61,62]. The pathogenesis of hMDS is still unclear; however, hematological improvement of blood counts after IST is the strongest indirect evidence of an immune-mediated BM growth suppression, as described in AA [61]. Oligoclonal expansion of CTLs is also frequent during hMDS with overexpression of FasL and TNF-related apoptosis-inducing ligand (TRAIL) on HSPCs [63,64,65,66,67,68,69]. Tregs are impaired in low-risk MDS, and Th22 and Th17 subsets are less represented in the peripheral blood, suggesting a derangement of immune responses in hMDS and low-risk MDS, which modifies cytokine composition in the BM niche (Figure 3A) [70,71,72,73,74,75].

Several cytokines, such as TNF-α, TRAIL, IFN-γ, Flice-like inhibitory protein (FLIP), TGF-β, or IL-17, are frequently increased in the sera of hMDS and low-risk MDS patients (Table 2) [61,76,77,78,79,80]. Low circulating IL-10 levels can be linked to decreased immunoregulatory activity of the immune system with enhancement of Th1 responses, ultimately leading to BM growth inhibition. IFN-γ in vitro blockade restores colony formation in hMDS and refractory anemia MDS. When compared with AA cytokine signature, several cytokines are significantly increased in the plasma of hMDS compared with that of AA, such as CCL5, CXCL5, CCL11, CXCL11, CCL3, CCL4, IL-1ra, and IL-6, while only TPO is decreased [26]. However, only TPO and CCL3 might be discriminatory in the differential diagnosis because of the minimal overlap of cytokine circulating concentrations between diseases [26]. hMDS show a greater homogeneity in cytokine profiling compared with NH-MDS, which are a heterogenous group of hematologic disorders with various clinical and molecular features. A recent meta-analysis has reported that TNF-α, IL-6, and IL-8 levels are significantly increased in MDS, regardless of stratification risk score based on the International Prognostic Scoring System (IPPS) [80,81,82,83], while several differences in cytokine levels are reported between low- and high-risk MDS. In particular, hMDS cytokine signature is more similar to that of low-risk MDS than that of high-risk MDS. Indeed, low-risk MDS show increased levels of circulating TNF-α, IFN-γ, TGFβ, IL-17, CXCL5, CCL5, CCL11, CD40L, EGF, and VEGF compared with high-risk MDS [26], with decreased levels of circulating IL-10 and CCL4 [61]. In the BM of MDS patients, IL-8, IP10, MCP-1, and IL-27 are significantly higher in both low- and high-risk MDS compared with healthy subjects, and levels increased during hypomethylating agent treatment [84]. In addition, MMP-1, MMP-9, and the inhibitor TIMP-2 levels are decreased in high-risk MDS compared with low-risk patients [85]. When comparing cytokine signature between hMDS and low-risk MDS based on the literature, five cytokines are commonly increased in the plasma of patients: TNF-α; IFN-γ; TGF-β; IL-17; CXCL5; CCL5; CCL11; CD40L; VEGF; and IL-6 (Figure 3B). Protein pathway analysis with STRING and Reactome databases [86,87] reveals that shared cytokines are mainly involved in cytokine and chemokine signaling in the immune system, TNF and IL-17 pathways, and infectious diseases (Figure 3C), suggesting common immunological derangement in low-risk and hypoplastic MDS pathogenesis.

## 4. Large Granular Lymphocyte Leukemia

Large granular lymphocyte (LGL) leukemia is a clonal lymphoproliferative disorder characterized by the presence of LGL with typical morphology and phenotype showing positivity for CD3, TCR αβ, CD8, CD16, CD5dim, CD45RA, and CD57; negativity for CD4, CD27, CD28, and CD45R0; and a phenotype similar to that of constitutively activated T-cells and terminal-effector memory T cells [88,89,90]. Lymphocytosis is frequent, and T-LGLs show clonality of the TCR repertoire and constitutive activation of STAT3b caused by somatic gain-of-function mutations. However, in some cases, differential diagnosis with other BMF syndrome is challenging because patients show lymphopenia without recurrent infections, transfusion-dependent anemia (10–30% of cases), thrombocytopenia (<25% of cases), or pure red cell aplasia (PRCA). In addition, AA, paroxysmal nocturnal hemoglobinuria (PNH), or MDS may co-exist with T-LGL leukemia [1,2,88]. On the other hand, AA patients might also have increased frequency of oligoclonal CD8^+^CD57^+^ effector memory T cells mimicking LGL clones [9]. Association with autoimmune diseases, such as rheumatoid arthritis, is also frequent in T-LGL leukemia patients [88].

**Figure 4:**
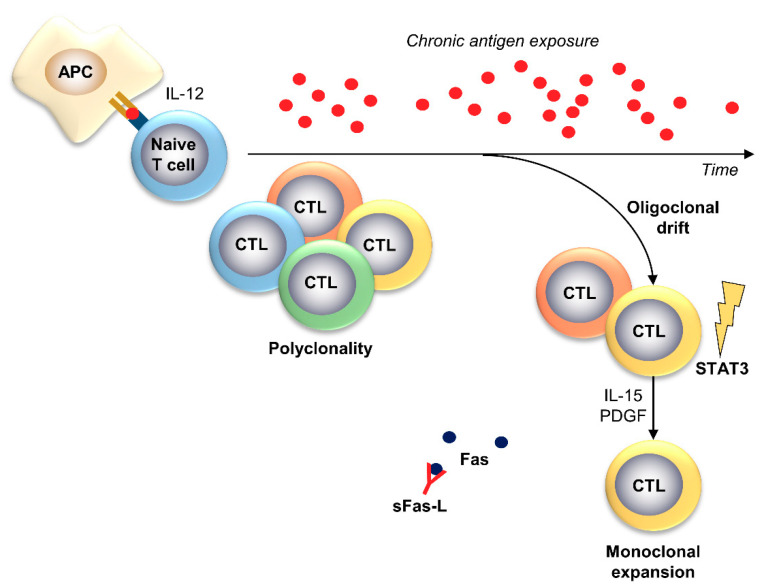
Pathophysiology of T-LGL leukemia. In T-LGL leukemia, an unknown chronic antigen causes the clonal drift of a T cell clonotype [88,91]. Acquisition of somatic mutations in STAT3 and overexpression of IL-15 and/or PDGF drive the monoclonal expansion of the leukemic clone [94,97]. Alterations in apoptosis pathways, such as inhibition of Fas-mediated apoptosis through binding of soluble Fas-ligand (sFas-L), also favor survival of the T-LGL clone [88,98,99,100].

The most accepted pathogenetic hypothesis is a clonal drift of a T cell population under chronic antigen exposure with a dramatic dysregulation of activation-induced cell death pathways (Figure 4). Several viral infections have been suggested to trigger the initial T cell activation, such as the human T-cell lymphotropic virus (HTLV) or the hepatitis C virus [88,91]; however, great heterogeneity of the TCR Vβ and Vα repertoires has been described, indicating that clonotypes are private to LGL leukemia because of the unlimited number of candidate epitopes in contrast to the limited number of epitopes in infectious diseases [9,92,93]. IL-15 and platelet-derived growth factors (PDGFs) play crucial roles in LGL clone expansion. IL-15 is a proinflammatory cytokine and the β and γ chains of the IL-2 receptor (CD122/CD132) form the IL-15 receptor for signaling transduction [94]. First evidence of the leukemogenic effects of IL-15 was described in 1994 in HTLV1-associated human T cell leukemia, where IL-15 levels were highly increased and anti-CD122 monoclonal antibody treatment reduced leukemic cell growth [95,96]. Similarly, in LGL leukemia, IL-15 is overexpressed, and chronic exposure of normal LGLs to IL-15 induces leukemic transformation with chromosomal and centrosome abnormalities [97].

Centrosome alterations leading to aneuploidy are frequently caused by overexpression of aurora kinases AurkA and AurkB, in which gene transcription is regulated by IL-15. Indeed, short-term cultures of LGLs in the presence of IL-15 show increased expression of MYC and ultimately of AURKA and AURKB, and hypermethylation of tumor suppressor genes mainly via DNMT3B induction [97]. Monoclonal LGL expansion is also driven by other two mechanisms: somatic STAT3B mutations and resistance to Fas/FasL-mediated apoptosis [88,98]. Soluble FasL (sFasL) is increased in the sera of LGL leukemia patients and acts as a decoy receptor blocking apoptotic events triggered by Fas [99,100]. Apoptotic inhibition is also mediated by increased activation of the PI3K/Akt signaling pathway via RANTES, IL-18, and MIP-1b at higher serum concentrations in LGL patients compared with healthy subjects [101,102]. Moreover, hyperactivation of NF-κB through TRAIL receptor activation can also lead to increased resistance to apoptosis in LGLs [103]. In addition, circulating levels of IFN-α2, IFN-γ, monocyte chemoattractant protein-1, epidermal growth factor, IL-6, IL-8, IL-10, IL-1β, IL-12p35, IL-1Ra, and MIP1-a are increased in the sera of LGL leukemia patients (Table 3) [104,105].

## 5. Paroxysmal Nocturnal Hemoglobinuria

PNH is a clonal non-malignant hematological disease characterized by the clinical triad of hemolytic anemia, BMF, and increased risk of thromboembolic events, and caused by somatic mutations in the X-linked phosphatidyl-inositol glycan class A (*PIG-A*) gene in HSCs [106,107]. Somatic mutations in *PIG-A* produce the lack of an important enzyme involved in the glycosylphosphatidyl inositol (GPI) anchor biosynthesis, thus proteins that need the GPI-anchor to correctly localize on the cell membrane cannot attach and exert their functions. Among all known GPI-anchored proteins, the lack of two complement-regulatory proteins, CD59 and CD55, determines an uncontrolled complement cascade activation, increasing the susceptibility of complement-mediated cell lysis [108]. Thrombophilia might be also related to the lack of urokinase-type plasminogen activator receptor (uPAR) on the cell surface with increased concentrations of its soluble form, leading to impairment in the fibrinolytic system [106]. However, HSCs harboring a *PIG-A* mutation do not have any proliferative advantages compared with normal cells, as small PNH clones can be found in healthy subjects; therefore, additional external events, such as BMF onset, should occur to induce disease development and progression [106,109]. Evidence shows the involvement of immune responses in triggering HSC destruction. To the best of our knowledge, few data are available on cytokine signature in PNH; however, plasma levels of TNF-α, TGF-β, and IFN-γ could be increased in PNH patients compared with healthy subjects [110]. The frequency of IFN-γ-producing lymphocytes is negatively correlated with circulating white blood cell and platelet counts [111], suggesting that IFN-γ can also act as a hematopoietic suppressor during PNH, as described in other BMF syndromes [112,113]. Meanwhile, IL-2-producing lymphocytes are decreased, probably because of the lack of GPI-anchored proteins involved in intracellular signaling transduction [111]. Circulating TNF-α levels might be increased and peripheral CD4^+^ memory T cells can have upregulation of genes involved in the TNF signaling pathway, such as TNFR and ATF2 [114]. Finally, oligoclonal expansion of CD8^+^ T cells can be also found in PNH patients as described in AA, and CDR3 sequences are shared between patients and healthy subjects, probably underlying a common epitope that triggers the autologous immune attach [115]. Noteworthy, one PNH-associated clonotype (CATSRTGGETQYF) was found in 11/12 AA patients and 8/9 healthy subjects at similar frequencies, confirming that PNH clones can be present in individuals without the disease and further proposing the clinical and biological overlap between AA and PNH [9].

## 6. Inherited BMF Syndromes

Inherited BMF syndromes consist of several clinical congenital entities caused by specific germline mutations and characterized by uni- or multi-lineage cytopenias and increased risk of developing MDS, AML, or a solid tumor [1,2,116]. These congenital disorders include FA, DKC, SDS, and Diamond–Blackfan anemia (DBA), and can be diagnosed in children and adults (aged 16 and older), especially for FA and DKC (up to 50% of cases), with a projected cumulative median survival age of 16–72 years [117]. Unlike immune-mediated BMF syndromes, inherited disorders are non-responsive to IST and hematopoietic stem cell transplantation remains the only curative therapeutic strategy for recovering from marrow failure [116,117,118].

### 6.1. Fanconi Anemia

FA is an autosomal or X-linked recessive disease characterized by malfunctioning of DNA repair mechanisms, leading to increased frequency of DNA double strand (dsDNA) breaks by DNA cross-linking agents, hypersensitivity to oxidative stress, and frequent chromosomal abnormalities [119,120,121]. Genetic alterations can occur over 17 different genes: A (FANCA), B (FANCB), C (FANCC), D1 (FANCD1/BRCA2), D2 (FANCD2), E (FANCE), F(FANCF), G (FANCG), I (FANCI/KIAA1794), J (FANCJ/BRIP1), L (FANCL), M (FANCM), N (FANCN/PALB2), P (FANCP/SLX4/BTBD12), O (FANCO/RAD51C), S (FANCS/BRCA1), and T (FANCT/UBE2T). These proteins play an essential role in DNA repair; that is, the core complex composed of FANCA, B, C, E, F, G, L, and M is an E3 monoubiquitin ligase and activates the ID complex (FANCD2 and FANCI) after DNA damage or replication stress is detected. After ubiquitination, the ID complex recruits FAN1 nuclease, FANCD1/BRCA2, FANCJ, and FANCN on the site of dsDNA break, and once correctly localized and stabilized, this complex associates with other DNA repair protein, such as RAD51 and BRCA1, and dsDNA breaks are repaired while the cell cycle is stopped [119,122,123]. The question, “why does the bone marrow fail in Fanconi anemia?” still has an unclear answer [124]. BMF seems to occur because of a progressive decline of CD34+ cells in the BM compartment, probably starting in the uterus; however, FA-deficient mice can develop various grades of BMF after treatment with cross-linking agents without showing the complete FA phenotype [125]. Indeed, unresolved DNA damage is needed for initiation of BMF. Evidence shows that the main genotoxic agents during FA are reactive oxygen species (ROS) and endogenous aldehydes; double knock-out mice for Aldh2, an enzyme that oxidizes acetaldehyde to acetate, and Fancd2 have developmental abnormalities and mostly died within 6 months of life because of acute lymphoblastic leukemia [126]. In FA, oxidative stress causes the accumulation of dsDNA breaks, leading to a progressive p53-dependent depletion of the HSC pool. Immune cells can also be affected; in particular, patients can show a decreased number of B and NK cells [127,128,129], and impairment in cytotoxic T cell and NK cell activities [118,129]. Immunoglobulin levels can be decreased in FA patients with severe BMF, especially IgG and IgM [128,129]. There is no specific cytokine signature in FA as few cytokines are found to be increased in the sera of patients, and the findings are discordant depending on disease severity. For example, Korthof et al. found increased serum levels of TGF-β, IL-6, and low soluble CD40 ligand, and no changes in IL-1β, IL-2, IL-4, IL-10, IL-13, IL-17, and IL-23 compared with healthy subjects [128]; while Justo and colleagues described higher plasma levels of IL-10 and no differences in TGF-β compared with controls [130]. The role of TNF-α and IFN-γ in FA development is still under investigation. Historically, TNF-α and IFN-γ have been proposed as BM growth inhibitors because of their functions in immune-mediated BMF syndrome pathogenesis [131]; however, these cytokines might play a different role in FA by pushing HSCs toward differentiation, and thus enhancing oxidative stress and DNA damage [124]. BM-activated T cells of FA patients have increased expression of TNF-α and IFN-γ; however, these findings were not confirmed by Matsui et al., who showed an increased susceptibility of peripheral monocytes to produce TNF-α, IL-6, and IL-1β in response to low dose lipopolysaccharide [118].

### 6.2. Diamond–Blackfan Anemia

DBA is characterized by physical abnormalities and macrocytic anemia with erythroid hypoplasia in the BM. In 25% of cases, modifications in transcription, splicing, or translation in genes encoding for ribosomal proteins, such as RPS19 and RPS24, are present with increased erythrocyte adenosine deaminase (eADA) activity [119,132]. Ribosomal proteins are essential in protein synthesis and also have extra-ribosomal functions, such as regulation of hematopoiesis and red blood cell maturation. Indeed, the expression of RPS19 is low in late stages of erythropoiesis compared with CD34^+^ HSCs, hinting a critical role of ribosomal proteins in regulation of protein synthesis in erythroid precursors [133]. In a del(5q) MDS model, Rps14 haploinsufficiency has been linked to increased innate immune responses and to a p53-dependent erythroid differentiation defect, and the heterozygous deletion of another ribosomal protein, Rps6, induces a DBA phenotype in a mouse model that can be rescued by inactivating p53 [134]. However, RPS14 or RPS6 inactivation has not been reported yet in DBA patients, suggesting that additional pathogenetic mechanisms are required for BMF development. Similar to DBA, more than 95% of SDS patients carry mutations in the SBDS (Shwachman–Bodian–Diamond syndrome) gene, mostly caused by gene conversion with the adjacent pseudogene. SBDS encodes for a protein involved in the 60S subunit ribosome formation [119]. Alterations in ribosomal functions lead to BMF in both DBA and SDS; however, clinical phenotypes are completely different underlying distinct extra-ribosomal functions of RPS proteins and SBDS. Indeed, SDS patients have physical abnormalities, malabsorption, and neutropenia, and the risk of solid tumor is not increased as in FA and DKC [132]. There are few studies investigating immune and cytokine levels in DBA and SDS; however, no significant alterations in immune responses are reported [118,127]. Indeed, serum immunoglobulin levels can be decreased, but within normal ranges, and no significant changes are described in circulating cytokines, including TNF-α and IFN-γ [118]. Peripheral lymphocytes and monocytes are lower in DBA and SDS patients compared with controls. In addition, after stimulation with phorbol 12-myristate 13-acetate and ionomycin, TNF-α and IFN-γ production by CD3^+^ T cells is decreased in DBA compared with healthy subjects and other inherited BMF syndromes, as well as TNF-α-producing CD14^+^ monocytes, while no alterations are reported in SDS [118].

### 6.3. Dyskeratosis Congenita

DKC, the first discovered telomerophaty, is characterized by skin hyperpigmentation, oral leukoplakia, and nail dystrophy, and patients lately have developed BMF, pulmonary fibrosis, and cancer. Mutations in nine different genes involved in telomere biology can be responsible for different clinical DKC phenotypes: DKC1, TERT, TERC, TINF2, WRAP53, NOP10, NHP2, CTC1, and RTEL1 [119,135]. The most frequent mutated genes are DKC1 on the X chromosome encoding for dyskerin; TRF1-interacting nuclear factor 2 (TINF2) encoding for the shelterin component TIN2; and heterozygous TINF2 mutations, which cause the most severe phenotype. About 10% of DKC patients carry mutations in TERT and TR, and rare autosomal recessive DKC are caused by mutations in telomerase accessory protein genes, such as NHP2, NOP10, and TCAB1 [136]. Disease manifestations can vary based on genetic alterations, and patients with mild symptoms or without physical alterations can receive a diagnosis of DKC only during adulthood when pulmonary fibrosis or aplastic anemia appears [132]. In the latter, the BM is hypocellular and aplastic, completely resembling AA [137], thus only screening for mutations in BMF-related genes can help clinicians in differential diagnosis. In addition, modifications in telomere biology can also be found in AA and worse BMF; however, mechanisms by which telomere attrition is triggered are different [137]. A gradual telomere loss is physiological with age as small portions of telomeres are lost during each cell division, regardless an optimal elongation by telomerase holoenzyme and shelterin complex [136]. In DKC and other telomeropathies, germline mutations in genes related to telomere repair lead to impairment in normal functions of telomerase or shelterin complex, and telomeres are not elongated correctly at each cell cycle, resulting in great telomere loss, apoptosis, and decreased HSC pool. In AA, telomere attrition might be linked to a replicative stress caused by the attempt of the BM to rescue the normal hemopoiesis [137]. Loss of HSC pool can also result in decreased circulating levels of B and T cells and monocytes [118]. Few studies have systematically investigated cytokine levels in DKC; however, only G-CSF, Flt3L (Flt3 ligand), and IP-10 can be increased in the sera of DKC patients with severe BMF, while RANTES can be lower than DKC patients with mild to moderate BMF or healthy subjects [127].

## 7. Therapy-Related MDS

MDS can be a de novo disease or arise after a previous chemo- or radiotherapy. In the latter, MDS is defined as therapy- or treatment-related MDS (tMDS) and is more frequently described in long-survivals of Hodgkin and non-Hodgkin lymphomas (NHL), acute lymphoblastic leukemia, sarcomas, and other solid tumors such as testicular cancer [138,139,140]. Incidence ranges from 0.8% to up to 24.3% in patients receiving autologous hematopoietic stem cell transplantation (HSCT) [139]. Known risk factors are a previous treatment with alkylating agents or radiation therapy identifying a specific clinical sub entity, or previous treatment with topoisomerase II inhibitors that recognized a different clinical entity as outlined by the World Health Organization [139,140]. Pathophysiology of tMDS can be linked to direct damage to the HSC genome; however, evidence shows the involvement of external factors and cytokines. For example, a prolonged administration of colony-stimulating factor (CSF) in NHL patients receiving chemotherapy is associated with an increased risk of tMDS development [141]. Radiation therapy can induce TNF-α production, leading to dyspoiesis, BM angiogenesis, and modifications in BM niche and stroma as described in de novo MDS [142]. Gene expression profiling of HSPCs obtained from tMDS patients who have received autologous (HSCT) has shown downregulation of genes involved in mitochondria and oxidative phosphorylation, ribosomes, proteasome, or cell cycle, with upregulation of genes involved in hematopoietic regulation, such as Hedgehog or HOX [143]. Increased susceptibility to DNA damage caused by impairment in mitochondrial oxidative phosphorylation and ROS elimination can augment genomic instability in HSPCs, ultimately leading to tMDS or AML.

## 8. Conclusions

BMF syndromes are characterized by hematopoietic failure and various grade of peripheral blood cytopenia(s); however, their pathogenesis varies even though a common immune signature could be identified [2,144]. In AA and hMDS, Th1 cells and CTLs are mainly responsible of the autologous BM destruction and release of proinflammatory cytokines, such as TNF-α and IFN-γ, causing BM growth inhibition directly or indirectly by sustaining autologous immune responses [2]. In T-LGL leukemia, hematopoietic failure is caused by BM infiltration of LGLs and release of proinflammatory cytokines, especially IL-15, which is a potent inhibitor of hemopoiesis [88]. In PNH, complement-mediated cell lysis is responsible for hemolytic anemia; however, increased circulating levels of TNF-α, TGF-β, and IFN-γ can be described [106,110]. Therefore, diagnostic and pathophysiologic overlaps among BMF syndromes might be translated into cytokine profiling similarities because several cytokines can be found to be augmented in different BMF syndromes, such as IL-1ra and IL-6, which can be increased in both hMDS and T-LGL leukemia, or IFN-γ, which is a common proinflammatory mediator involved in immune response polarization and BM growth inhibition. Whether cytokines are drivers or passengers in BMF development is still an open question. Indeed, prolonged in vitro exposure to TNFα and IFNγ can induce senescence through increased oxidative stress, reactive oxygen species (ROS) production, and DNA damage, as also recently described in DBA [145,146]. Oxidative stress and DNA damage are generated by IL1β and TGFβ persistent stimulation. Senescent cells are physiologically removed by immune cells; in turn, lymphocytes can induce cancer growth arrest and senescence through Th1 cytokines, in a “dog-biting-tail” mechanism [147,148]. However, whether this process is also involved in BMF development is still unclear [117].

BMF cytokines signatures are pivotal not only for a better understanding of disease pathophysiology, but also for identification of novel diagnostic and prognostic biomarkers and candidate therapeutic targets. Unfortunately, because of the complex cross-talk between HSPCs, stromal cell, and immune cells, and of the intricate mixture of released cytokines present in the BM niche, the use of a single anti-IL or anti-TNF agent in the BMF syndromes has shown little efficacy in improvement of blood counts [61]. However, specific changes in cytokine signatures might identify candidate biomarkers of responsiveness to therapies, thus improving clinical management of patients by early identification of poor responders or disease progression.

## Figures and Tables

**Table 1:** Deregulated cytokines in acquired aplastic anemia (AA).

	ILs	Chemokines	IFNs/TNFs	Growth Factors	Others
Increased	IL-2	CXCL10 CCL20	IFN-γ TNFα	G-CSF TPO EPO	GDF-15 sST2
	IL-8				
	IL-12				
	IL-17A				
	IL-18				
	IL-21				
	IL-23				
Decreased	IL-33 IL-35	CCL5		EGF VEGF	CD40L
		CCL11			SELL
		CCL17			DKK1
		CXCL5			c-Mpl
		CXCL11			Hepcidin
No changes	IL-1Ra IL-6	CCL2		HGF (or slightly reduced)	
		CCL3			S100A8
		CCL4			S100A9
		CXCL9			S100A8/A9
		CXCL11			

Abbreviations. ILs, interleukins; IFNs, interferons; TNFs, tumor necrosis factors; CCL, CC chemokine ligands; CXCL, C-X-C motif chemokine; G-CSF, granulocyte colony-stimulating factor; TPO, thrombopoietin; EPO, erythropoietin; EGC, epidermal growth factor; VEGF, vascular endothelial growth factor; HGF, hepatocyte growth factor; GDF, growth differentiation factor; SELL, L-selectin; DKK1, Dickkopf-related protein 1; c-Mpl, thrombopoietin receptor.

**Table 2:** Deregulated cytokines in hypoplastic myelodysplastic syndrome (hMDS).

	ILs	Chemokines	IFNs/TNFs	Growth Factors	Others
Increased		CCL3		VEGF	
	IL-1ra	CCL4	IFN-γ		CD40L
	IL-6	CCL5	TNFα		TGF-β
	IL-17	CCL11	TRAIL		FLIP
		CXCL5			
		CXCL11			
Decreased	IL-10			TPO	

Abbreviations. ILs, interleukins; IFNs, interferons; TNFs, tumor necrosis factors; CCL, CC chemokine ligands; CXCL, C-X-C motif chemokine; TRAIL, TNF-related apoptosis-inducing ligand; VEGF, vascular endothelial growth factor; TPO, thrombopoietin; TGF, tumor growth factor; FLIP, FLICE-like inhibitory protein.

**Table 3:** Deregulated cytokines in large granular lymphocyte (LGL) leukemia.

	ILs	Chemokines	IFNs/TNFs	Growth Factors	Others
Increased	IL-1β	CCL2	IFN-γ IFN-α2	PDGF EGF	
	IL-1ra				
	IL-6				RANTES
	IL-8				MIP-1α
	IL-10				MIP-1β
	IL-12p35				sFas-L
	IL-15				B2M
	sIL-15Rα				
	IL-18				
Decreased					FLIP

Abbreviations. ILs, interleukins; IFNs, interferons; TNFs, tumor necrosis factors; CCL, CC chemokine ligands; CXCL, PDGF, platelet-derived growth factor, EGF, epidermal growth factor; RANTES, regulated on activation, normal t cell expressed and secreted; MIP, macrophage inflammatory protein; sFas-L, soluble Fas ligand; B2M, beta-2 microglobulin; FLIP, FLICE-like inhibitory protein.

## Data Availability

Not applicable.
